# Association among *Helicobacter pylori* Infection, Tooth Loss, and Heavy Medal Exposure in a Chinese Rural Population

**DOI:** 10.3390/ijerph19084569

**Published:** 2022-04-11

**Authors:** Jun Yan, Honglong Zhang, Zenan Hu, Xuan Zhang, Jingping Niu, Bin Luo, Haiping Wang, Xun Li

**Affiliations:** 1 Department of General Surgery, The First Hospital of Lanzhou University, Lanzhou 730000, China; ldyy_yanj@lzu.edu.cn; 2 Key Laboratory of Biotherapy and Regenerative Medicine of Gansu Province, Lanzhou 730000, China; wanghp21@lzu.edu.cn; 3 The First School of Clinical Medine, Lanzhou University, Lanzhou 730000, China; hlzhang21@lzu.edu.cn; 4 Department of Digestive Diseases, The First Hospital of Lanzhou University, Lanzhou 730000, China; huzn@lzu.edu.cn; 5 School of Stomatology, Northwest Minzu University, Lanzhou 730000, China; 285112048@xbmu.edu.cn; 6 Institute of Occupational and Environmental Health, School of Public Health, Lanzhou University, Lanzhou 730000, China; niujingp@lzu.edu.cn (J.N.); luob@lzu.edu.cn (B.L.)

**Keywords:** heavy metal, cadmium, lead, tooth loss, *Helicobacter pylori*, blood analysis, ^14^C-urea breath test

## Abstract

Previous research suggests that heavy metals may be associated with increased susceptibility to *Helicobacter pylori* infection. This study investigated the effect of heavy metal exposure (Pb and Cd) on tooth loss and *H. pylori* infection in a Chinese rural population, who live near a mining and smelting area. Blood samples were collected from the study participants to estimate the lead (Pb) and cadmium (Cd) exposure levels. *H. pylori* infection was analyzed using the ^14^C-urea breath test, and the number of missing teeth (MT), filled teeth (FT), and missing or filled teeth (MFT) were counted by conducting a physical examination. Regression analysis was used to assess the difference between *H. pylori*-positive and -negative individuals in the MT, FT, and MFT groups, adjusting for confounders. The *H. pylori* infection prevalence was higher in individuals in the high Cd or high Pb groups than that in the low Cd or low Pb groups (*p* < 0.05). In addition, greater numbers of FT and MFT were observed in individuals in the high Pb group than those in the low Pb group (*p* < 0.05). We further found 8.7% (95% CI, 2.8–23.8%, *p* = 0.017) of the effect of the high BPb level on *H. pylori* infection risk could be statistically explained by FT using amediation analyses in adjusted models, and 6.8% (95% CI, 1.6–24.8%, *p* = 0.066) by MFT. Furthermore, FT and MFT were significantly associated with increased risk for *H. pylori* infection (odds ratio (OR) = 4.938, 95% confidence interval (CI): 1.125–21.671; OR = 3.602, 95% CI: 1.218–10.648, respectively). Pb and Cd exposure may be associated with tooth loss and increased susceptibility to *H. pylori* infection, and tooth loss may be an independent risk factor for *H. pylori* infection.

## 1. Introduction

Increased susceptibility to *H. pylori* infection may be related to human exposure to heavy metals, such as lead (Pb) and cadmium (Cd). Studies have shown that there are differences in the concentration of serum elements, such as zinc [1], cobalt [2], and magnesium [1], between individuals infected with *H. pylori* and those uninfected. Krueger and Wade showed that elevated blood lead (BPb) and cadmium (BCd) levels were significantly associated with *H. pylori* seropositivity, suggesting that the suppression effects of Pb and Cd toxicity on the immune response may be account for increased susceptibility to *H. pylori* infection [3].

Recent epidemiological evidence and the occurrence of *H. pylori* in food and water confirm that *H. pylori* may be a foodborne pathogen [4,5,6,7]; therefore, oral problems (including teeth problems) are likely to be an important intermediate link between Pb and Cd exposure and *H. pylori* infection. Previous studies have shown that environmental Pb and Cd exposure is associated with dental caries and reduced bone density [8,9]. Pb and Cd that enter the mouth through food are incorporated into hydroxyapatite crystals, leading to the development of hydroxyapatite crystal deposition disease (HADD), a disease of uncertain etiology characterized by deposition of hydroxyapatite crystals in the shoulder and other arthrosis [10]. They replace calcium ions in teeth, causing enamel hypoplasia and increased abrasion and even tooth loss [11,12]. Browar et al. showed that Cd causes loss of alveolar bone in a rodent model of long-term Cd poisoning [13], and further found that higher Cd content in human mandibles is associated with poorer periodontal scores [14]. Similarly, Pb exposure has been reported to affect the gingival health of children and increases the risk of dental caries [15,16]. However, it remains unknown as to whether Pb and Cd increase the susceptibility to *H. pylori* infection among residents in Pb- and Cd-exposed areas by causing tooth loss. Conflicting results have been reported on the association between dental problems, including missing teeth (MT), filled teeth (FT), missing or filled teeth (MFT), and *H. pylori* infection. Alag et al. found that the number of MT does not seem to be associated with gastric or oral *H pylori* positivity [17]. In contrast, Shimoyama et al. found that *H. pylori* infection was associated with a decreased risk of tooth loss in healthy Japanese men [18]. Therefore, it is necessary to further study the association between tooth loss and *H. pylori* infection in rural populations exposed to heavy metals.

Given that the relationship between *H. pylori* prevalence and dental problems in populations living in heavy metal-polluted areas has not been previously investigated, we analyzed the BPb and BCd levels in middle-aged and elderly residents living near a mining and smelting area in northwest China, which is dominated by Pb and Cd exposure, and then we assessed dental problems and *H. pylori* infection in this population. This study is of great value as it comprehensively evaluates the association between Pb and Cd exposure and dental problems, including the number of MT, FT, MFT, and *H. pylori* infections.

## 2. Materials and Methods

### 2.1. Study Areas

**Figure 1:**
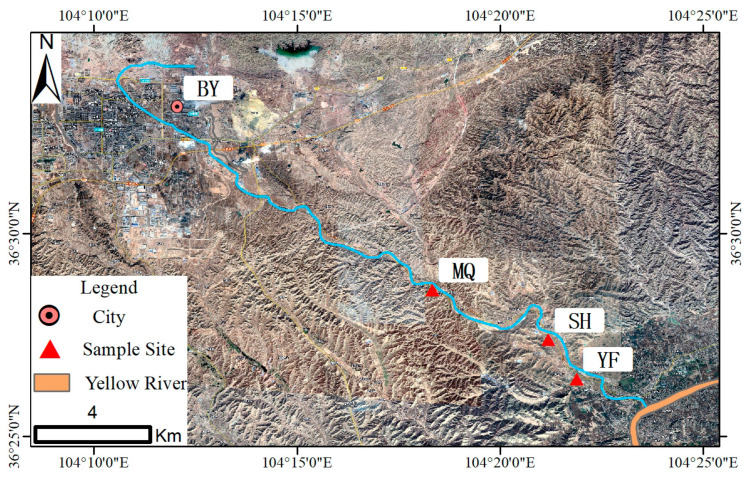
Map of the study area. MQ, SH, and YF represent MQ village, SH village, and YF village, respectively. BY represents BY city, which is an important metal mining and smelting base.

The study area was located in the Dongdagou watershed in northwest China, which is known as an important non-ferrous metal mining and smelting base in China since the 1950s [19]. The metal mining, smelting, and processing industries have greatly promoted societal and economic development. However, local farmers have used industrial sewage to irrigate the surrounding farmland owing to the limited availability of water sources, resulting in heavy metal pollution of agricultural soils and crops. Previous reports have shown that the soil in this watershed contains high concentrations of heavy metals (including Pb and Cd), which have been the main pollutants in farmland soils, and the Cd content (1.27–2.51 mg/kg), in particular, has been found to be 2.12–4.18 times the allowable maximum content (MC) of the national secondary standard [20,21]. Although the soil Pb content did not reach the allowable MC (350.00 mg/kg), the Pb concentration (34.85 mg/kg) in orchard soil was slightly but significantly higher than the soil background value (18.80 mg/kg) in NW China [20,22]. Moreover, a correlation analysis further confirmed that the Cd and Pb contents in corn grains mainly originated from contaminated soils [23]. High pollutant levels in food pose a threat to human health through the food chain. Due to environmental protection concerns, the Chinese government launched a project for the comprehensive prevention and control of heavy metal pollution in 30 priority districts nationwide in 2015, of which Dongdagou Watershed in NW China ranked first [22]. We selected three villages in the Dongdagou watershed in NW China closest to the non-ferrous metal mining and smelting areas as the research area. Among all the villages, MQ village is the village closest to the mineral-polluted locations in the upper and middle reaches of the Dongdagou stream, and the SH and YF villages are in turn close to the MQ village (Figure 1). These three areas are dominated by the Han nationality, with similar customs, culture, and living habits.

### 2.2. Study Population

We conducted a cross-sectional study within the Dongdagou-Xinglong (DDGXL) cohort, which was established in 2015. In this cohort study, we carried out environmental heavy metal testing, as well as resident heavy metal exposure and health status testing. Since then, health information and resident sample databases have been established. The inclusion and exclusion criteria have also been reported in previous studies [24].

**Figure 2:**
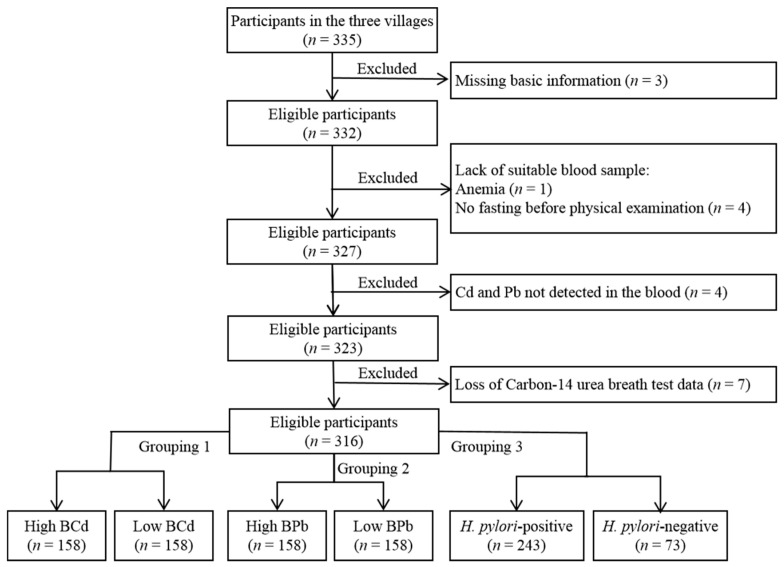
Flow chart of study participants who met the inclusion criteria and were included in this study (*n* = 316). Blood cadmium (BCd) and blood lead (BPb) levels were recoded into categorical variables classified as high (≥50th percentile), or low (<50th percentile).

We adopted the form of a census to conduct door-to-door surveys in the aforementioned three villages, and all individuals meeting the inclusion and exclusion criteria were included in this study (*n* = 335 natives aged 40–69 years). A detailed questionnaire, including demographic information, number of MT, FT, and MFT, gastric history, and habit of cigarette smoking, alcohol consumption, drinking tea, and salt intake was administered to all participants. However, 19 individuals were not included in the final analysis due to missing basic information (*n* = 3), lack of suitable blood samples (*n* = 5), lack of heavy metal detection in the samples (*n* = 4), and loss of carbon-14 urea breath test data (*n* = 7). Hence, 316 subjects (199 women and 117 men) were included in the final analysis (Figure 2). The study was approved by the ethics committee of the First Hospital of Lanzhou University, according to the guidelines of the Declaration of Helsinki (ethical codes: LDYYLL2015-0027, LDYYLL2020-103).

### 2.3. Sample Collection 

A strict sampling protocol was followed throughout the study. A total of 10 mL of peripheral venous blood was collected from each subject by well-trained nurses. Approximately 5 mL of blood was collected in an anticoagulant tube, gently mixed upside down, and dispensed into 1.5 mL centrifuge tubes, which were transported back to the biological sample bank on dry ice on time and stored at −80 °C. The remaining 5 mL of blood was collected in a coagulation tube, centrifuged at 3000 rpm for 15 min to obtain serum, and stored at −80 °C for later use [24].

### 2.4. Heavy Metal Determination in Whole Blood

A previously published protocol for determination of blood metal concentrations was followed [24]. BCd and BPb measurements were performed using inductively coupled plasma-mass spectrometry (ICP-MS, PerkinElmer, Waltham, MA, USA) at the Chongqing Prevention and Treatment Center for Occupational Diseases. We used the calibration standards (PerkinElmer, Waltham, MA, USA) to control the calibration ranges of blood samples, and analytical quality controls were applied using certified standard reference materials from Seronorm^TM^ Trace Elements Whole Blood (SERO AS, Billingstad, Norway). The limit of detection was 0.08 μg/L for BCd, and 0.11 μg/L for BPb, and All calibration standard checks were satisfactory.

### 2.5. Carbon-14 Urea Breath Test

The subjects stopped taking antibiotics and bismuth-containing products, as well as using pump inhibitors for over 1 month and over 2 weeks prior to the test, respectively. All subjects who fasted overnight were tested for *H. pylori* using the ^14^C-urea breath test, which was performed by trained clinicians following the manufacturer’s protocols (Headway, Shenzhen, China). Briefly, subjects were given 1 ^14^C capsule with 20 mL of cold drinking water in advance, after sitting still for 25 min. Subsequently, they exhaled directly into the gas cylinder until the liquid in the gas cylinder changed from pink to colorless, or continued to exhale for 3 min. Then, a 4.5 mL diluted scintillation solution was added to the gas cylinder, capped and tightened, and placed in the liquid scintillator for detection. The results were expressed as counts per minute (CPM) and graded (0 = negative for *H. pylori* infection, CPM ≤ 100, and 1 = positive for *H. pylori* infection, CPM > 100), as suggested by the manufacturer [25].

### 2.6. Statistical Analysis

Database management and statistical analysis were performed using SPSS (version 24.0; SPSS Inc., Chicago, IL, USA). All graphics were plotted using GraphPad Prism 8 (GraphPad Software, Chicago, IL, USA). Normality was evaluated using the Kolmogorov–Smirnov test. Quantitative data with normal distribution (age, height, weight, BMI, and waist circumference) are shown as the mean ± standard deviation (SD), whereas the median (interquartile range) is shown for data that were not normally distributed (BCd and BPb). Qualitative data, including gender, education level, income, occupation, cigarette smoking, alcohol consumption, tea drinking, gastric history, salt intake, MT, FT, and MFT, are shown as frequencies (rates). One-way analysis of variance (ANOVA), the Kruskal–Wallis rank sum test, and the Chi-squared test were used to compare the data from the three villages in the study area. We calculated the median BCd and BPb levels, which were then recoded into categorical variables classified as high (≥50th percentile), or low (<50th percentile). The Student’s t-test or Chi-squared test was used to analyze the different exposure levels and subgroups with different grades of *H. pylori* infection. Multiple linear regression was used to analyze the association between oral problems (including MT, FT, and MFT) and *H. pylori* infection. Logistic models were used to show the relationship between *H. pylori* infection and the number of MT, FT, and MFT. In addition, we calculated the mediation proportion and its 95% CI [26] using the publicly available % Mediate macro (https://www.hsph.harvard.edu/donna-spiegelman/software/mediate/, accessed on 7 February 2022). The mediation proportion quantified the extent to which the association between Pb exposure and *H. pylori* infection risk is mediated through Pb exposure relative to dental problems (including FT and MFT). Differences were considered statistically significant at a *p* value of <0.05.

## 3. Results

### 3.1. Characteristics of the Study Population in the Different Villages

A total of 316 participants were divided into the YF (*n* = 99), SH (*n* = 91), and MQ (*n* = 126) villages based on the distance between the villages where residents lived and the mining and smelting area. Table 1 shows that no significant differences in basic information, such as gender, age, weight, waist circumference, education level, occupation, alcohol consumption, tea drinking, or salt intake, were observed among the subjects living in these three villages. However, the body mass index (BMI) was higher in individuals from the YF village than that in individuals from the SH and MQ villages (*p* < 0.01). Furthermore, the subjects living in the YF village had a lower height, family income, and smoking prevalence than those in the other two villages (*p* < 0.01 or *p* < 0.05). Moreover, the exposure levels (including BCd and BPb levels), *H. pylori* positivity rate, and the numbers of FT and MFT of subjects living in the MQ village were significantly higher than those of subjects living in the other two villages (*p* < 0.01 or *p* < 0.05).

### 3.2. Association of H. pylori Infection and Dental Problems with Heavy Metal Exposure Levels

Based on the detected differences in heavy metal levels, *H. pylori* infection, and dental problems in individuals from the different villages, we next analyzed whether *H. pylori* infection and dental problems are related to the levels of heavy metal exposure. Table 2 shows the *H. pylori* positivity rate and dental problems of subjects with different exposure levels (high BCd, low BCd, high BPb, and low BPb). The *H. pylori* positivity rate was significantly associated with the exposure levels. In particular, the *H. pylori* positivity rate in the high BCd group was increased by 9.4% when compared to that of the low BCd group (*p* < 0.05), while that of the high BPb group was also higher than that of the low BPb group (83.5 vs. 70.3%, *p* < 0.01). In addition, subjects in the high BPb group had higher numbers of FT and MFT when compared to those in the low BPb group (*p* < 0.05), and subjects in the high BCd group also showed similar trends, but the difference was not statistically significant. We further analyzed the mediation effect of FT or MFT on the relationship between BPb concentration and *H. pylori* infection using mediation analyses in adjusted models, and the results showed 8.7% (95% CI, 2.8–23.8%, *p* = 0.017) of the effect of the high BPb level on *H. pylori* infection risk could be statistically explained by FT, and 6.8% (95% CI, 1.6%–24.8%, *p* = 0.066) by MFT. 

### 3.3. Association of H. pylori infection with Dental Problems 

**Figure 3:**
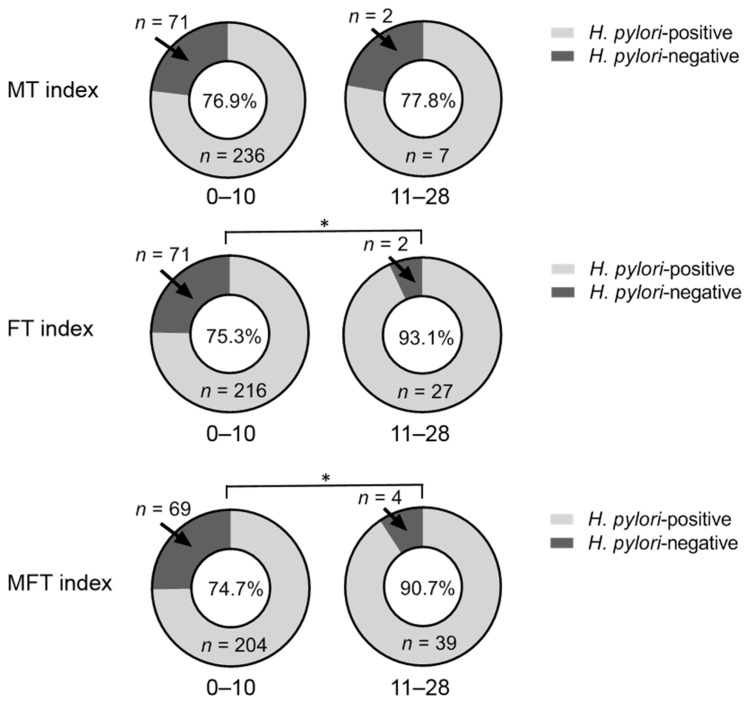
Comparison of the *Helicobacter pylori (H. pylori)* positivity rates associated with the different dental problems. The Chi-squared test was used to assess the differences in the *H. pylori*-positive rates associated with different dental problems. The subjects in the 11–28 missing teeth (MT), 11–28 filled teeth (FT), and 11–28 missing or filled teeth (MFT) groups had a higher *H. pylori* positivity rate than those in the 0–10 MT, 0–10 FT, and 0–10 MFT groups. *H. pylori* positivity rates are shown in the pie graph center. * Indicates statistically significant differences between the *H. pylori*-positive and *H. pylori*-negative groups (*p* < 0.05).

We found that heavy metal exposure (BCd and BPb) may be an important factor for the increase in the number of MT and *H. pylori* positivity rate among residents living near mining and smelting areas. Furthermore, Figure 3 compares the differences in the *H pylori* positivity rates associated with different dental problems. The *H. pylori* positivity rate of subjects with 11–28 FT increased by 17.8% when compared to that of individuals with 0–10 FT (*p* < 0.05), and the *H. pylori* positivity rate of subjects with 11–28 MFT was also higher than that of individuals with 0–10 MFT (90.7 vs. 74.7%, *p* < 0.05). Additionally, higher *H pylori* positivity rates were observed in the 11–28 MT group when compared to those of the 0–10 MT group, but the difference was not statistically significant.

### 3.4. Association between the H. pylori Infection and Dental Problems

Considering the age, gender, BMI, cigarette smoking, alcohol consumption, and salt intake of study participants, we analyzed the effect of dental problems (the numbers of MT, FT, and MFT) on *H. pylori* infection using a logistic model (Table 3). After adjusting for confounders, FT and MFT remained as *H. pylori* infection risk factors (*p* = 0.034, *p* = 0.020). The OR of subjects with 11–28 FT was 4.956 (95% CI: 1.126–21.813) when compared to that of individuals with 0–10 FT. Similar results were observed in the MFT subgroups, and the OR of subjects with 11–28 MFT was 3.617 (95% CI: 1.220–10.723) when compared to that of individuals with 0–10 MFT. Additionally, the relationships between *H. pylori* infection and dental problems in the full multiple linear regression model are shown in Table S1.

## 4. Discussion

Due to its proximity to mining and smelting areas, the Dongdagou watershed in northwestern China has become an area threatened by heavy metals. Although great environmental investigation and management efforts have been made in the area for decades [27,28], no health surveys, especially with regard to dental problems and *H. pylori* infection, have been conducted on local residents until the initiation of our screening work. In this study, we investigated the impact of heavy metal exposure (Pb and Cd) on dental problems and *H. pylori* infection in a Chinese rural population. Our data show a higher prevalence of *H. pylori* infection among subjects chronically exposed to Pb and Cd. Furthermore, the number of MT and FT was associated with the prevalence of *H. pylori* infection, which may partially explain the above findings. Moreover, the risk of *H. pylori* positivity among residents exposed to Pb and Cd increased with increasing numbers of MT. This risk is expected to be reduced by filling teeth. 

*H. pylori* infection remains a major public health concern worldwide. The prevalence of *H. pylori* infection in this study (76.9%) was higher than the average prevalence of *H. pylori* infection in China, as previously reported. Although, different studies have indicated that the variation in the prevalence of *H. pylori* in various populations depends on lifestyle habits and geographic area [25,29,30]. Hooi et al. reported that the average prevalence of *H. pylori* infection in residents of China was 55.8% in a meta-analysis of 22 cohort studies [31], whereas recent declines in *H. pylori* prevalence were observed due to rising standards of living and improved sanitation. We believe that the high level of *H. pylori* infection in the present study population may be related to living in heavy metal-polluted areas. Studies have reported that the BCd and BPb levels, and in particular the Pb levels, were significantly associated with *H. pylori* seroprevalence among participants under 13 years of age and American adult women [3,32]. Moreover, as indicated by another study, even a small increase in the BPb levels may increase the risk of *H. pylori* infection [33]. These findings are consistent with ours showing that subjects with high BCd and BPb levels had an elevated *H. pylori* positivity rate. 

*H. pylori* infection is related to many factors, such as age, occupation, smoking, and diet [34,35]. *H. pylori* is a foodborne pathogen, and we suspect that the high prevalence of *H. pylori* in people exposed to Pb and Cd may be associated with dental problems. Furthermore, we analyzed the number of MT, FT, and MFT in the study population and found that residents in the MQ village (high Pb and Cd exposure levels) had higher numbers of FT and MFT, especially in the high BPb group, which was similar to the findings of previous studies. Alomary et al. found that the concentrations of Pb and Cd in carious teeth were significantly higher than those in non-carious teeth [36]; Shiue et al. also reported that the study population with bone loss around the mouth and loose teeth presented with higher levels of Cd [37]. Mechanistically, Cd is extremely nephrotoxic and osteotoxic, and the osteotoxicity of Cd is a secondary effect of renal injury, resulting from the imbalance of Ca and P metabolism caused by kidney damage, which manifests as accelerated bone loss. Pb can directly produce osteotoxic effects, causing osteoblasts to undergo degeneration, while increasing osteoclast formation and activity [9,38]. Teeth and bones have the same chemical composition (hydroxyapatite). Long-term exposure to Pb and Cd can cause periodontal disease and tooth loss [39].

Finally, we analyzed the relationship between *H. pylori* infection and dental problems in people exposed to Pb and Cd, and found that the greater the numbers of FT and MFT are, the stronger the correlation between FT, MFT, and *H. pylori* infection is. Previous studies have not reached a consistent conclusion on the relationship between *H. pylori* infection and dental problems [17,37,40,41]. For example, Zahedi et al. reported an association between gastric infection with *H. pylori* and the The Decayed, Missing, and Filled Teeth (DMFT) index [41]. However, Alag et al. did not find an association between the number of MTs and decayed teeth and the positivity of oral *H. pylori* [17]. Our results are similar to those of Pearce et al. [42]. The risk of positivity for *H. pylori* infection increased with the increasing number of MFTs, but after filling teeth, this risk was greatly reduced, suggesting the importance of filling teeth. Although FT is a risk factor for *H. pylori* infection when compared with normal teeth, filling teeth can reduce the high risk of *H. pylori* infection caused by tooth loss. In short, the association between tooth loss and *H. pylori* infection may provide an indirect theoretical basis for exploring the effects of Pb and Cd exposure on *H. pylori* infection.

It remains unclear why tooth loss is a risk factor for increased susceptibility to *H. pylori* infection. It is generally believed that tooth loss is mainly caused by oral problems, such as periodontitis, tooth decay, and loose teeth [43], which are associated with behavioral and lifestyle factors, including poor oral hygiene and excessive intake of sugary foods [44,45], leading to increased risk of tooth loss and *H. pylori* infection. Additionally, tooth loss could be a surrogate marker for past bacterial load on teeth and perhaps for the presence of endogenous bacteria in general, but larger bacterial deposits in turn are more likely to be due to poor dental hygiene, so poor dental hygiene may increase the chance of tooth loss and *H. pylori* infection [46]. Furthermore, the microbial community composed of bacteria, fungi, viruses, and archaea living in the oral cavity is defined as the oral microbiome. They interact and exist in a biofilm state, known as the oral biofilm, which in turn affects the host’s health and disease status [47]. Tooth loss is likely to break the balance of oral biofilms, leading to systemic inflammation and eugenic H pylori, which enters the stomach with food and affects the health of the body. These findings point out a novel direction for future research.

There are some limitations to the present study. First, only the BCd and BPb levels of residents in heavy metal polluted areas were detected in this study, but heavy metals in blood are usually present in the body as mixtures, so more heavy metals need to be detected in future studies. Second, in addition to its association with dental problems, the relationship between *H. pylori* infection and other factors has not been investigated. A larger number of subjects exposed to Pb and Cd should be included in future studies on the association between *H. pylori* infection and age, gender, BMI, occupation, education level, and eating habits, to further verify the findings of this study. Third, this study did not measure *H. pylori* in the oral cavity, so we cannot directly answer the question of whether tooth loss provides an environment for *H. pylori* to grow, and the data of the existing clinical statistical analysis cannot allow us to derive the mechanism of tooth loss in *H. pylori*. Additionally, the cross-sectional design of the present study does not enable us to allow us to explore the time trend of heavy metal exposure, *H. pylori* infection, and dental problems. In vivo experiments and in vitro validation are still required to clarify the causal relationship and specific mechanism of tooth loss and *H. pylori*. Finally, it may be more meaningful to construct a collaborative study involving both dentists and gastroenterologists to explore the relationship between complex oral problems and *H. pylori* infection.

## 5. Conclusions

In summary, this is the first study to conduct heavy metal exposure assessment and investigation of *H. pylori* infection and the associated risk factors in local residents in a heavy metal-polluted area in northwestern China. The results of this study indicated a high prevalence of *H. pylori* infection and increased numbers of MT, FT, and MFT among residents closest to the mining and smelting areas; the same phenomenon was also observed in residents in high Pb or high Cd exposure groups. Furthermore, based on the current data, we speculate that the high prevalence of *H. pylori* infection among residents exposed to Pb and Cd may be associated with tooth loss, and that filling teeth can reduce the risk of *H. pylori* infection. These findings provide new insights into the high prevalence of *H. pylori* infection in heavy metal-exposed areas, and may contribute to developing new strategies to prevent *H. pylori* infection, which is vital for the health of residents in these areas.

## Figures and Tables

**Table 1:** Characteristics of the study population in the different villages.

	YF Village (*n* = 99)	SH Village (*n* = 91)	MQ Village (*n* = 126)	*p* Value
**Demographic characteristics**				
Height (Wu et al.)	159.68 ± 6.53	163.49 ± 8.29	162.03 ± 8.84	0.004 *
Weight (kg)	62.81 ± 9.33	62.39 ± 11.16	61.43 ± 10.78	0.596
BMI (kg/m^2^)	24.66 ± 3.61	23.30 ± 3.51	23.29 ± 2.84	0.003 *
Waist circumference (Wu et al.)	83.87 ± 9.37	84.13 ± 9.59	85.25 ± 8.95	0.491
Age (years)	56.30 ± 11.39	55.70 ± 6.18	56.45 ± 6.96	0.802
Gender				0.226
Male	30 (30.3)	35 (38.5)	52 (41.3)	
Female	69 (69.7)	56 (61.5)	74 (58.7)	
Education level				0.626
Illiteracy	32 (32.3)	25 (27.5)	32 (25.4)	
Primary school	26 (26.3)	19 (20.9)	28 (22.2)	
Middle school	32 (32.3)	34 (37.4)	45 (35.7)	
High school	9 (9.1)	13 (14.3)	21 (16.7)	
Family income/person/year (¥)				0.001 *
<1000	12 (12.8)	10 (11.2)	2 (1.7)	
1000–3000	24 (25.5)	26 (29.2)	19 (15.8)	
3000–6000	26 (27.7)	28 (31.5)	40 (33.3)	
6000–10,000	27 (28.7)	18 (20.2)	38 (31.7)	
>10,000	5 (5.3)	7 (7.9)	21 (17.5)	
Occupation				0.683
Farmer	92 (92.9)	85 (93.4)	114 (90.5)	
No farmer	7 (7.1)	6 (6.6)	12 (9.5)	
Cigarette smoking				0.050 *
Yes	24 (24.2)	30 (33.0)	50 (39.7)	
No	75 (75.8)	61 (67.0)	76 (60.3)	
Alcohol consumption				0.058
Yes	5 (5.1)	6 (6.6)	17 (13.5)	
No	94 (94.9)	85 (93.4)	109 (86.5)	
Tea drinking				0.939
Yes	37 (37.4)	35 (38.5)	50 (39.7)	
No	62 (62.6)	56 (61.5)	76 (60.3)	
Salt intake				0.218
High-salt (>6 g/day)	9 (9.1)	16 (12.7)	16 (17.6)	
Normal (≤6 g/day)	90 (90.9)	110 (87.3)	75 (82.4)	
Gastric history				0.010 *
Yes	12 (12.1)	2 (2.2)	18 (14.3)	
No	87 (87.9)	89 (97.8)	108 (85.7)	
**Carbon-14 urea breath test**				
*H. pylori* infection				0.001 *
Negative	36 (36.4)	18 (19.8)	19 (15.1)	
Positive	63 (63.6)	73 (80.2)	107 (84.9)	
**Dental problems**				
MT index				0.242
0–10	94 (94.9)	90 (98.9)	123 (97.6)	
11–28	5 (5.1)	1 (1.1)	3 (2.4)	
FT index				0.012 *
0–10	93 (93.9)	87 (95.6)	107 (84.9)	
11–28	6 (6.1)	4 (4.4)	19 (15.1)	
MFT index				0.048 *
0–10	87 (87.9)	84 (92.3)	102 (81.0)	
11–28	12 (12.1)	7 (7.7)	24 (19.0)	
**Toxic trace metals**				
BCd (ng/mL)	0.42 (0.08–1.52)	2.92 (0.08–5.91)	4.82 (2.81–7.79)	<0.001 *
BPb (ng/mL)	21.32 (10.92–27.71)	22.44 (12.42–30.21)	44.34 (34.99–56.81)	<0.001 *

*H. pylori*: *Helicobacter pylori*; MT: missing teeth; FT: filled teeth; MFT: missing or filled teeth; BCd: cadmium in blood; BPb: lead in blood. Height, weight, BMI, waist circumference, and age are shown as the mean ± standard deviation (SD) and were compared using one-way analysis of variance (ANOVA). Other continuous data are shown as medians (interquartile range) and were compared using Kruskal-Wallis rank sum test. Chi-square test was used for categorical data, which are shown as frequencies (rates). * indicates significant differences among subjects in the YF, SH, and MQ villages.

**Table 2:** Association of *H. pylori* infection and dental problems with heavy metal exposure levels.

	High BCd (*n* = 158)	Low BCd (*n* = 158)	*p* Value	High BPb (*n* = 158)	Low BPb (*n* = 158)	*p* Value
**Carbon-14 urea breath test**						
*H. pylori* infection			0.031*			0.004 *
Negative	29 (18.4)	44 (27.8)		26 (16.5)	47 (29.7)	
Positive	129 (81.6)	114 (72.2)		132 (83.5)	111 (70.3)	
**Dental problems**						
MT index			0.251			0.500
0–10	155 (98.1)	152 (96.2)		154 (97.5)	153 (96.8)	
11–28	3 (1.9)	6 (3.8)		4 (2.5)	5 (3.2)	
FT index			0.121			0.003 *
0–10	140 (88.6)	147 (93.0)		136 (86.1)	151 (95.6)	
11–28	18 (11.4)	11 (7.0)		22 (13.9)	7 (4.4)	
MFT index			0.372			0.024 *
0–10	135 (85.4)	138 (87.3)		130 (82.3)	143 (90.5)	
11–28	23 (14.6)	20 (12.7)		28 (17.7)	15 (9.5)	

*H. pylori: Helicobacter pylori*; MT: missing teeth; FT: filled teeth; MFT: missing or filled teeth; BCd: cadmium in blood; BPb: lead in blood. Chi-squared test was used for categorical data, which are shown as frequencies (rates). * indicates significant differences between subjects in the high and low BCd/BPb groups.

**Table 3:** ORs (95% CIs) for *H. pylori* infection by dental problems using logistic regression analysis.

	Model 1		Model 2	
	OR (95%CI)	*p* Value	OR (95%CI)	*p* Value
MT index				
0–10	reference		reference	
11–28	1.209 (0.251–5.821)	0.813	1.218 (0.248–5.994)	0.808
FT index				
0–10	reference		reference	
11–28	4.437 (1.029–19.129)	0.046 *	4.938 (1.125–21.671)	0.034 *
MFT index				
0–10	reference		reference	
11–28	3.298 (1.137–9.563)	0.028 *	3.602 (1.218–10.648)	0.020 *

MT: missing teeth; FT: filled teeth; MFT: missing or filled teeth; CI: confidence interval; OR: odds ratio. Model 1 adjusted with nothing; Model 2 adjusted with age, gender, BMI, cigarette smoking, alcohol consumption, and salt intake. All data are shown as OR (95%CI) and were analyzed using logistic regression analysis. * *p* < 0.05.

## Data Availability

The datasets used and/or analysed during the current study are available from the corresponding author on reasonable request.

## Supplementary Materials

The following supporting information can be downloaded at: https://www.mdpi.com/article/10.3390/ijerph19084569/s1, Table S1: Association between *H. pylori* infection and dental problems using multiple linear regression analysis.

## References

[1] Ztürk N., Kurt N., Özgeriş F.B., Baygutalp N.K., Tosun M.S., Bakan N. (2015). Serum Zinc, Copper, Magnesium and Selenium Levels in Children with *Helicobacter pylori* Infection. Eurasian J. Med..

[2] Hu A., Li L., Hu C., Zhang D., Wang C., Jiang Y. (2018). Serum Concentrations of 15 Elements Among *Helicobacter pylori*-Infected Residents from Lujiang County with High Gastric Cancer Risk in Eastern China. Biol. Trace Elem. Res..

[3] Krueger W.S., Wade T.J. (2016). Elevated blood lead and cadmium levels associated with chronic infections among non-smokers in a cross-sectional analysis of NHANES data. Environ. Health.

[4] Leja M., Grinberga-Derica I., Bilgilier C., Steininger C. (2019). Review: Epidemiology of *Helicobacter pylori* infection. Helicobacter.

[5] Pina-Pérez M.C., González A., Moreno Y., Ferrús M.A. (2019). *Helicobacter pylori* Detection in Shellfish: A Real-Time Quantitative Polymerase Chain Reaction Approach. Foodborne Pathog. Dis..

[6] Ng C.G., Loke M.F., Goh K.L., Vadivelu J., Ho B. (2017). Biofilm formation enhances *Helicobacter pylori* survivability in vegetables. Food Microbiol..

[7] Rusiñol M., Hundesa A., Cárdenas-Youngs Y., Fernández-Bravo A., Pérez-Cataluña A., Moreno-Mesonero L. (2020). Microbiologial contamination of conventional and reclaimed irrigation water: Evaluation and management measures. Sci. Total Environ..

[8] Pradeep K.K., Hegde A.M. (2013). Lead exposure and its relation to dental caries in children. J. Clin. Pediatr. Dent..

[9] Ma Y., Ran D., Shi X., Zhao H., Liu Z. (2021). Cadmium toxicity: A role in bone cell function and teeth development. Sci. Total Environ..

[10] Goller S.S., Hesse N., Dürr H.R., Ricke J., Schmitt R. (2021). Hydroxyapatite deposition disease of the wrist with intraosseous migration to the lunate bone. Skelet. Radiol..

[11] Foxman B., Kolderman E., Salzman E., Cronenwett A., Gonzalez-Cabezas C., Neiswanger K., Marazita M.L. (2019). Primary teeth microhardness and lead (Pb) levels. Heliyon.

[12] Chen H.-L., Fang J., Chang C.-J., Wu T.-F., Wang I.-K., Fu J.-F., Huang Y.-C., Yen J.-S., Weng C.-H., Yen T.-H. (2021). Environmental Cadmium Exposure and Dental Indices in Orthodontic Patients. Healthcare.

[13] Browar A.W., Koufos E.B., Wei Y., Leavitt L.L., Prozialeck W.C., Edwards J.R. (2018). Cadmium Exposure Disrupts Periodontal Bone in Experimental Animals: Implications for Periodontal Disease in Humans. Toxics.

[14] Browar A.W., Leavitt L.L., Prozialeck W.C., Edwards J.R. (2019). Levels of Cadmium in Human Mandibular Bone. Toxics.

[15] Tort B., Choi Y.-H., Kim E.-K., Jung Y.-S., Ha M., Song K.-B., Lee Y.-E. (2018). Lead exposure may affect gingival health in children. BMC Oral Health.

[16] Wu Y., Jansen E.C., Peterson K.E., Foxman B., Goodrich J.M., Hu H., Solano-González M., Cantoral A., Téllez-Rojo M.M., Martinez-Mier E.A. (2018). The associations between lead exposure at multiple sensitive life periods and dental caries risks in permanent teeth. Sci. Total Environ..

[17] Alagl A.S., Abdelsalam M., El Tantawi M., Madi M., Aljindan R., Alsayyah A. (2019). Association between *Helicobacter pylori* gastritis and dental diseases: A cross-sectional, hospital-based study in Eastern Saudi Arabia. J. Periodontol..

[18] Shimoyama T., Higuchi H., Matsuzaka M., Chinda D., Nakaji S., Fukuda S. (2013). *Helicobacter pylori* infection is associated with a decreased risk of tooth loss in healthy Japanese men. Jpn. J. Infect. Dis..

[19] Li Y., Wang S., Nan Z., Zang F., Sun H., Zhang Q., Huang W., Bao L. (2019). Accumulation, fractionation and health risk as-sessment of fluoride and heavy metals in soil-crop systems in northwest China. Sci Total Environ..

[20] Liu B., Ma X., Ai S., Zhu S., Zhang W., Zhang Y. (2016). Spatial distribution and source identification of heavy metals in soils under different land uses in a sewage irrigation region, northwest China. J. Soils Sediments.

[21] Nan Z., Zhao C. (2000). Heavy Metal Concentrations in Gray Calcareous Soils of Baiyin Region, Gansu Province, P.R. China. Water Air Soil Pollut..

[22] Liu B., Ai S., Zhang W., Huang D., Zhang Y. (2017). Assessment of the bioavailability, bioaccessibility and transfer of heavy metals in the soil-grain-human systems near a mining and smelting area in NW China. Sci. Total Environ..

[23] Zang F., Wang S., Nan Z., Ma J., Zhang Q., Chen Y., Li Y. (2017). Accumulation, spatio-temporal distribution, and risk assessment of heavy metals in the soil-corn system around a polymetallic mining area from the Loess Plateau, northwest China. Geoderma.

[24] Zhang H., Yan J., Niu J., Wang H., Li X. (2022). Association between lead and cadmium co-exposure and systemic immune in-flammation in residents living near a mining and smelting area in NW China. Chemosphere.

[25] Zhang F., Pu K., Wu Z., Zhang Z., Liu X., Chen Z., Ye Y., Wang Y., Zheng Y., Zhang J. (2020). Prevalence and associated risk factors of *Helicobacter pylori* infection in the Wuwei cohort of north-western China. Trop. Med. Int. Health.

[26] Hertzmark E., Pazaris M., Spiegelman D. (2018). The SAS MEDIATE Macro. Harvard T.H. Chan School of Public Health. https://www.hsph.harvard.edu/donna--spiegelman/software/mediate/.

[27] Li Y., Wang Y.-B., Gou X., Su Y.-B., Wang G. (2006). Risk assessment of heavy metals in soils and vegetables around non-ferrous metals mining and smelting sites, Baiyin, China. J. Environ. Sci..

[28] He B., Wang W., Geng R., Ding Z., Luo D., Qiu J., Zheng G., Fan Q. (2020). Exploring the fate of heavy metals from mining and smelting activities in soil-crop system in Baiyin, NW China. Ecotoxicol. Environ. Saf..

[29] Li M., Sun Y., Yang J., de Martel C., Charvat H., Clifford G.M. (2020). Time trends and other sources of variation in Helico-bacter pylori infection in mainland China: A systematic review and meta-analysis. Helicobacter.

[30] Wang W., Jiang W., Zhu S., Sun X., Li P., Liu K., Liu H., Gu J., Zhang S. (2019). Assessment of prevalence and risk factors of *helicobacter pylori* infection in an oilfield Community in Hebei, China. BMC Gastroenterol..

[31] Hooi J., Lai W.Y., Ng W.K., Suen M., Underwood F.E., Tanyingoh D. (2017). Global Prevalence of *Helicobacter pylori* In-fection: Systematic Review and Meta-Analysis. Gastroenterology.

[32] Fong P., Wang Q.T. (2021). Protective effect of oral contraceptive against *Helicobacter pylori* infection in US adult females: NHANES 1999–2000. Epidemiol. Infect..

[33] Park W.-J., Kim S.-H., Kang W., Ahn J.-S., Cho S., Lim D.-Y., Kim S., Moon J.-D. (2019). Blood lead level and *Helicobacter pylori* infection in a healthy population: A cross-sectional study. Arch. Environ. Occup. Health.

[34] Zhu H.-M., Li B.-Y., Tang Z., She J., Liang X.-Y., Dong L.-K., Zhang M. (2020). Epidemiological investigation of *Helicobacter pylori* infection in elderly people in Beijing. World J. Clin. Cases.

[35] Szaflarska-Popławska A., Soroczyńska-Wrzyszcz A. (2019). Prevalence of *Helicobacter pylori* infection among junior high school students in Grudziadz, Poland. Helicobacter.

[36] Alomary A., Al-Momani I.F., Obeidat S.M., Massadeh A.M. (2012). Levels of lead, cadmium, copper, iron, and zinc in deciduous teeth of children living in Irbid, Jordan by ICP-OES: Some factors affecting their concentrations. Environ. Monit. Assess..

[37] Shiue I. (2015). Urinary heavy metals, phthalates, phenols, thiocyanate, parabens, pesticides, polyaromatic hydrocarbons but not arsenic or polyfluorinated compounds are associated with adult oral health: USA NHANES, 2011–2012. Environ. Sci. Pollut. Res..

[38] Chang L., Shen S., Zhang Z., Song X., Jiang Q. (2018). Study on the relationship between age and the concentrations of heavy metal elements in human bone. Ann. Transl. Med..

[39] Kakei M., Sakae T., Yoshikawa M. (2013). Combined effects of estrogen deficiency and cadmium exposure on calcified hard tissues: Animal model relating to itai-itai disease in postmenopausal women. Proc. Jpn. Acad. Ser. B.

[40] Zheng Y., Liu M., Shu H., Chen Z., Liu G., Zhang Y. (2014). Relationship between oral problems and *Helicobacter pylori* infection. Arch. Oral Biol..

[41] Zahedi L., Jafari E., Parizi M.T., Shafieipour S., Abbasi M.H.B., Moghadam S.D., Zahedi M.J. (2017). The Association between Oral Hygiene and Gastric Pathology in Patients with Dyspepsia: A Cross-Sectional Study in Southeast Iran. Middle East J. Dig. Dis..

[42] Pearce M.S., Steele J.G., Campbell D.I., Thomas J.E. (2005). Tooth Loss and *Helicobacter pylori* Seropositivity: The Newcastle Thousand Families Cohort Study at Age 49–51 Years. Helicobacter.

[43] Kinane D.F., Stathopoulou P.G., Papapanou P.N. (2017). Periodontal diseases. Nature reviews. Nat. Rev. Dis. Primers.

[44] Kotsakis G.A., Chrepa V., Shivappa N., Wirth M., Hébert J., Koyanagi A., Tyrovolas S. (2017). Diet-borne systemic inflammation is associated with prevalent tooth loss. Clin. Nutr..

[45] Melo P., Marques S., Silva O.M. (2017). Portuguese self-reported oral-hygiene habits and oral status. Int. Dent. J..

[46] Stolzenberg-Solomon R.Z., Dodd K.W., Blaser M.J., Virtamo J., Taylor P.R., Albanes D. (2003). Tooth loss, pancreatic cancer, and *Helicobacter pylori*. Am. J. Clin. Nutr..

[47] Radaic A., Kapila Y.L. (2021). The oralome and its dysbiosis: New insights into oral microbiome-host interactions. Comput. Struct. Biotechnol. J..

