# Supplementary material for: Association among Helicobacter pylori Infection, Tooth Loss, and Heavy Medal Exposure in a Chinese Rural Population
Source: Int J Environ Res Public Health. 2022 Apr 11;19(8):4569. doi: 10.3390/ijerph19084569 (PMC9025748; doi:10.3390/ijerph19084569)
Supplement: Supplementary file 1 [file ijerph-19-04569-s001.zip › ijerph-1609230-supplementary.pdf]

Association among *Helicobacter pylori* Infection, Tooth Loss, and Heavy Medal Exposure in a Chinese Rural Population

Jun Yan [1,2,3,†], Honglong Zhang [3,†], Zenan Hu [4], Xuan Zhang [5], Jingping Niu [6], Bin Luo [6], Haiping Wang [2,3] and Xun Li [1,2,3,*]

[1] Department of General Surgery, The First Hospital of Lanzhou University, Lanzhou 730000, China; ldyy_yanj@lzu.edu.cn
[2] Key Laboratory of Biotherapy and Regenerative Medicine of Gansu Province, Lanzhou 730000, China; wanghp21@lzu.edu.cn
[3] The First School of Clinical Medine, Lanzhou University, Lanzhou 730000, China; hlzhang21@lzu.edu.cn
[4] Department of Digestive Diseases, The First Hospital of Lanzhou University, Lanzhou 730000, China; huzn@lzu.edu.cn
[5] School of Stomatology, Northwest Minzu University, Lanzhou 730000, China; 285112048@xbmu.edu.cn
[6] Institute of Occupational and Environmental Health, School of Public Health, Lanzhou University,
Lanzhou 730000, China; niujingp@lzu.edu.cn (J.N.); luob@lzu.edu.cn (B.L.)
* Correspondence: lix@lzu.edu.cn; Tel: +86-13993138612
† These authors contributed equally to this work.

Table S1. Association between *H. pylori* infection and dental problems using multiple linear regression analysis.

	**Model 1**		**Model 2**	
	**β (95%CI)**	***p* Value**	**β (95%CI)**	***p* Value**
MT index				
0–10	reference		reference	
11–28	0.032 (−0.235 to 0.299)	0.814	0.033 (−0.240 to 0.360)	0.811
FT index				
0–10	reference		reference	
11–28	0.178 (0.018–0.339)	0.030 *	0.195 (0.030–0.360)	0.021 *
MFT index				
0–10	reference		reference	
11–28	0.160 (0.024–0.295)	0.021 *	0.172 (0.033–0.311)	0.015 *

MT: missing teeth; FT: filled teeth; MFT: missing or filled teeth; CI: confidence interval. Model 1 adjusted with nothing; Model 2 adjusted with BMI, gastric history, cigarette smoking, and alcohol consumption. All data are shown as β Coefficient (95%CI) and were analyzed by using multiple linear regression. * $p < 0.05$.
